# Stress and Psychosocial Distress Scale with Blunted Oscillatory Dynamics Serving Abstract Reasoning

**DOI:** 10.1155/2024/4720803

**Published:** 2024-04-23

**Authors:** Mikki Schantell, Ryan Glesinger, Anna T. Coutant, Hannah J. Okelberry, Jason A. John, Sarah M. Dietz, Seth D. Springer, Yasra Arif, Tony W. Wilson

**Affiliations:** ^1^Institute for Human Neuroscience, Boys Town National Research Hospital, Boys Town, NE, USA; ^2^College of Medicine, University of Nebraska Medical Center (UNMC), Omaha, NE, USA; ^3^Department of Pharmacology & Neuroscience, Creighton University, Omaha, NE, USA

## Abstract

**Background:**

Chronic stress is associated with a multitude of psychopathological disorders that share similar alterations in neural dynamics and symptomatology. Applying the National Institute of Mental Health's Research Domain Criteria (RDoC) framework, we probed the stress-diathesis model by identifying how a transdiagnostic psychosocial distress index representing high-dimensional patterns of stress-related aberrations was coupled to the neural oscillatory dynamics serving abstract reasoning.

**Methods:**

The sample consisted of 69 adults (mean age = 44.77 years, SD = 13.66) who completed the NIH Toolbox Emotion Battery (NIHTB-EB) and a matrix reasoning task during magnetoencephalography (MEG). A transdiagnostic psychosocial distress index was computed using exploratory factor analysis with assessments from the NIHTB-EB. Whole-brain correlations were conducted using the resulting psychosocial distress index for each oscillatory response, and the resulting peak voxels were extracted for mediation analyses to assess the degree to which neural oscillatory activity mediates the interplay between perceived stress and psychosocial distress.

**Results:**

We found that elevated psychosocial distress was associated with blunted oscillatory alpha/beta and gamma responses in key cortical association regions. Further, we found that only alpha/beta activity in the right superior temporal sulcus partially mediated the relationship between perceived stress and psychosocial distress.

**Conclusions:**

The present study is among the first to couple perceived stress and psychosocial distress with alterations in oscillatory activity during a matrix reasoning task. These findings illuminate the relationship between perceived stress and neural alterations associated with psychopathology.

## 1. Introduction

Stress is inextricably linked to a host of psychiatric disorders such as depression, anxiety, and suicidal thoughts and behaviors. In fact, stress has been identified as a primary determinant in predicting the later emergence of psychopathology and psychosocial distress. Psychosocial distress refers to the emotional and psychological suffering experienced by individuals in response to various social and psychological stressors such as isolation, bullying, trauma, major life changes, and chronic stress [1–3]. The stress-diathesis model can be particularly useful in conceptualizing psychosocial distress, as the model integrates cognitive, emotional, behavioral, and social predispositions to form a diathesis, which acts as a risk factor for future psychopathology. However, the diathesis alone is not sufficient to produce psychosocial distress, which requires potentiating factors to confer pathology [4]. Vulnerability to stress poses a substantial risk of psychosocial distress in those with a higher level of the diathesis, so much so that even minor stressors can lead to psychosocial distress, while those who have a lower level of diathesis may seldom experience psychosocial distress regardless of the degree of stress they encounter [4]. Childhood adversity in particular has repeatedly been found to increase the risk for psychiatric disorders in adolescence and adulthood, especially depression and suicide [5–7]. However, not all exposed individuals will develop these conditions following childhood adversity, suggesting there is a varying degree of diathesis present across individuals [4].

The National Institute of Mental Health's Research Domain Criteria (RDoC) framework may prove to be a promising avenue for advancing the field's current understanding of psychosocial distress by enabling the integration of cognitive, social, emotional, behavioral, and neurophysiological measures [8, 9] into a transdiagnostic psychosocial distress index to quantify high dimensional patterns of risk that cut across traditional indices of psychiatric symptoms. There are many factors that have been associated with psychopathology including aggression, anxiety, depression, social withdrawal, poor peer relationships, chronic stress, and executive dysfunction [10, 11]. Specifically, prolonged exposure to stress has been shown to disrupt the physiological mechanisms that regulate responses to potential threats [12], which can be observed through distinct alterations in behaviors (e.g., greater reactivity to aversive stimuli and reduced reactivity to passive stimuli [13–15]), dysregulation in the hypothalamic-pituitary-adrenal (HPA) axis [16, 17], elevated systemic inflammation [18], and changes in neural circuits serving central executive, fear, and reward networks [19, 20], thereby conferring greater risk to the development of psychiatric disorders such as depression and anxiety. Thus, it is critical to investigate psychosocial distress through a multifaceted lens, and such conceptual indices directly enable this.

Fluid intelligence (G*f*) is broadly defined as the ability to problem-solve in novel situations, learn new skills, and adapt to changing environments [21], and it is crucial for higher-order cognitive abilities such as abstract reasoning and executive function. Executive function, which encompasses a range of cognitive processes that serve goal-directed behavior, is known to utilize the prefrontal cortices (PFC) to help direct thoughts, behaviors, and emotions [22]. While healthy PFC functioning is important for adequate G*f* performance [23, 24], the parietofrontal integration theory of intelligence (P-FIT [15]) suggests that distributed network-level interactions between prefrontal and parietal cortices support G*f*.

Prior work has identified the key neural oscillations involved in G*f,* which include theta, alpha, and gamma oscillations in frontoparietal regions [25–30], with some studies showing that the strength of oscillations in these regions scales with greater cognitive demands and effort [31, 32]. Further, oscillatory responses in the PFC and parietal cortices have been shown to support more optimal performance in tasks involving G*f* [27, 28], thus underscoring the critical role oscillations play in the long-range communication and integration of the distributed networks serving these higher-order cognitive processes [32–34]. Despite mounting evidence separately supporting the role of stress in executive dysfunction and the breakdown of the neural dynamics serving G*f,* it remains unknown how stress impacts the neural oscillatory dynamics serving G*f,* and further, how the stress-related breakdown of these systems relates to psychosocial distress and psychopathology.

Thus, the goal of the present study was to identify how psychosocial distress affects the neural oscillatory markers serving G*f* using dynamic functional mapping with MEG. First, we examined the impact of higher psychosocial distress on the oscillatory dynamics serving abstract reasoning, which is closely tied to G*f*. We then assessed the degree to which perceived stress was related to neural function in the brain regions associated with psychosocial distress. We hypothesized that adults with elevated psychosocial distress would have blunted oscillatory activity in such brain regions, including the prefrontal, parietal, and temporal cortices, and that these regions would also be related to participants' levels of perceived stress.

## 2. Methods and Materials

### 2.1. Participants

Eighty-one cognitively normal adults between the ages of 20 and 66 years old (mean age: 46.04 years) were selected from a larger project (NIH R01-MH116782) based on their completion of the abstract reasoning task during MEG. We did not perform a power analysis to determine the sample size for this particular study, as our aim was to utilize all available data to examine possible relationships between psychosocial distress and neural oscillations serving abstract reasoning. Of note, the sample size was much larger than what is typical for studies using MEG. Exclusion criteria included any medical illness affecting CNS function; any self-reported history of neurological or major psychiatric disease (e.g., stroke, Alzheimer's disease, Parkinson's disease, epilepsy, schizophrenia, bipolar disorder, autism, current major depressive disorder, and posttraumatic stress disorder) diagnosed by a neurologist, psychiatrist, or clinical psychologist; reliance on external medical devices (e.g., pacemaker); history of head trauma resulting in loss of consciousness for more than five minutes; current pregnancy; illicit substance use; the use of medications that may interfere with neural functioning (e.g., anticonvulsants, antipsychotics, and barbiturates); and the MEG Center's standard criteria for ferromagnetic materials (e.g., participants must be free of excessive dental work and unremovable metallic implants/jewelry). All demographic data were obtained via self-report from participants during the intake process. The University of Nebraska Medical Center's Institutional Review Board reviewed and approved this investigation. Each participant provided written informed consent following a detailed description of the study.

### 2.2. Psychosocial Distress Index and Perceived Stress

**Figure 1:**
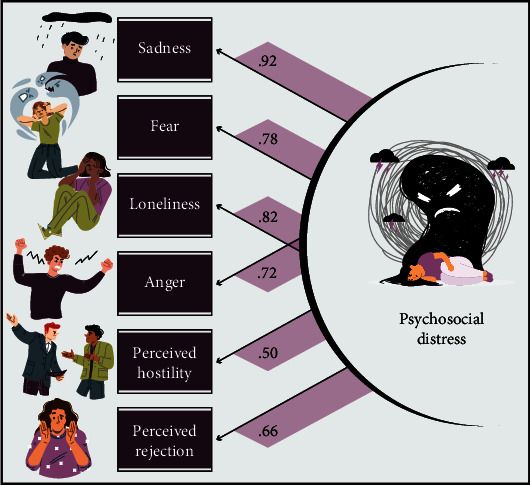
Modeling of the psychosocial distress factor. The factors contributing to the psychosocial distress index were derived from an exploratory factor analysis (EFA) and included *T*-scores of the following six measures of the NIH Toolbox Emotion Battery with their corresponding factor loadings: sadness, fear, loneliness, anger, perceived hostility, and perceived rejection.

To index psychosocial distress in the present sample, we conducted an exploratory factor analysis (EFA) using the maximum likelihood extraction method with a varimax rotation to define a latent variable of psychosocial distress using a compilation of metrics that are known to contribute to overall mental health and distress [10, 11]. We used *T*-scores from six measurements included in the NIH Toolbox Emotion Battery: anger affect, fear affect, sadness, loneliness, perceived hostility, and perceived rejection (Figure 1). All measures had moderate-to-high factor loadings (*λ* > 0.50) and converged onto one factor that had an eigenvalue of 3.77, which accounted for 62.78% of the variance. This model was used to define a continuous latent variable for which a psychosocial distress index score was extracted per participant, which uses the standardized (i.e., *z*-scores) observed values for each item included in the final factor and weighted by a regression coefficient [35]. Missing values were excluded using listwise deletion. Modeling was completed using SPSS (version 25). Higher values were indicative of greater psychosocial distress. Further, we used *T*-scores from the perceived stress measure of the NIH Toolbox Emotion Battery to evaluate participants' perceptions of how unpredictable and uncontrollable their lives are.

### 2.3. Abstract Reasoning Task Paradigm

**Figure 2:**
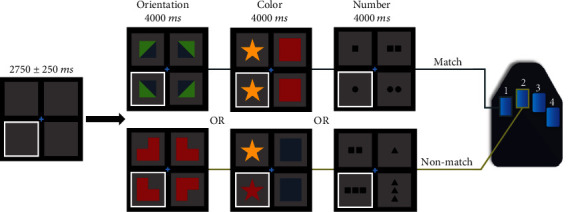
Abstract reasoning task paradigm. Participants were presented with an empty grid of gray boxes for 2500 to 3000 ms with either the left or right bottom square highlighted by a white border to indicate the location of the upcoming target. Complex images then populated each of the four squares within the grid for 4000 ms. Participants indicated whether the image in the highlighted square correctly completed the pattern in the grid by responding via button press (i.e., right index finger for matching patterns, 60 trials; right middle finger for nonmatching patterns, 60 trials). Match and nonmatch trials were presented in a pseudorandomized order for the duration of the task. Participants performed well on the task, with a mean accuracy of 84.70% (SD = 8.5%) and a mean reaction time of 1991.10 ms (SD = 294.6 ms).

Participants completed a nonprogressive abstract reasoning task adapted from the classic Raven's Progressive Matrices [36, 37]. Participants were shown a centrally presented fixation cross in a 2 × 2 grid for a period of 2500 to 3000 ms (Figure 2), with either the bottom left or bottom right box highlighted by a white border. An array of four complex figures was then presented in each of the four boxes within the 2 × 2 grid for 4000 ms. Participants were instructed to respond to whether the complex figure in the highlighted box accurately completed the 2 × 2 matrix based on the color, shape, and/or orientation of the patterns in the other three boxes. Participants responded by pressing a button with their right index finger if the highlighted figure correctly completed the matrix (i.e., match), or by pressing a button with their right middle finger if the highlighted figure did not correctly complete the matrix (i.e., nonmatch). There were 120 trials, equally split and pseudorandomized between correct and incorrect matrix completions. The task took approximately 14 minutes to complete.

### 2.4. MEG Data Acquisition

Functional MEG data were collected using a MEGIN MEG system (Helsinki, Finland) equipped with 306 sensors (204 planar gradiometers, 102 magnetometers) using a 1 kHz sampling rate and an acquisition bandwidth of 0.1-330 Hz in a one-layer magnetically shielded room with active shielding engaged. Prior to MEG acquisition, four coils were attached to the participant's head and localized along with fiducial and scalp surface points using a three-dimensional (3D) digitizer (FASTRAK, Polhemus Navigator Sciences, Colchester, Vermont). Once the participants were positioned for MEG recording, an electric current with a unique frequency label (e.g., 322 Hz) was fed to each of the four coils, thus inducing a measurable magnetic field which enabled each coil to be localized in reference to the MEG sensor array throughout the recording session.

### 2.5. MEG and MRI Processing

MEG and MRI data processing closely followed previously reported pipelines [27, 28, 38]. The structural MRI data were aligned parallel to the anterior and posterior commissures and transformed into standardized space. MEG data were subjected to environmental noise reduction and corrected for head motion using the signal space separation method with a temporal extension [39]. Only data from the 204 planar gradiometers were used for further analysis. All MEG and MRI data were further processed in BESA (research: version 7.1; MRI: version 3.0; statistics: version 2.1). Cardiac and ocular artifacts were removed from the MEG data using signal space projection (SSP; [40]), and this correction was accounted for during source analysis.

### 2.6. MEG Time-Frequency Transformation

The continuous magnetic time series was then filtered with a 60 Hz notch filter. Epochs were 6500 ms, with the baseline extending from -1800 to -800 ms prior to visual stimulus onset. Only trials with correct responses were considered for further analysis. Epochs containing artifacts were rejected using a fixed threshold method that was set per participant and supplemented with visual inspection. Briefly, in MEG, the raw signal amplitude is strongly affected by the distance between the brain and the MEG sensor array, as the magnetic field strength falls off sharply as the distance from the current source (i.e., brain) increases. To account for this source of variance across participants, as well as other sources of variance, we used an individualized threshold based on the signal distribution for both amplitude and gradient to reject artifacts. The average amplitude threshold across all participants was 1376.95 (SD = 702.27) fT/cm, the average gradient threshold was 336.47 (SD = 312.26) fT/(cm⁣^∗^ms), and an average of 109.90 (SD = 8.25) trials out of the original 120 were used for further analysis. The number of trials included in the final MEG analyses was not significantly associated with psychosocial distress (*r* = −0.07, *p* = 0.787) or perceived stress (*r* = −0.03, *p* = 0.551).

### 2.7. Sensor-Level Statistics

We then transformed the artifact-free epochs into the time-frequency domain (resolution: 2 Hz, 25 ms) using complex demodulation [41, 42]. Each sensor's spectral power estimations were averaged over trials to produce time-frequency plots of mean spectral density, which were then normalized by the baseline power of each respective bin, calculated as the mean power from -1800 to -800 ms. The time-frequency windows for subsequent source imaging were identified using a stringent two-stage statistical approach that utilized paired-sample *t*-tests against baseline on each pixel in the spectrogram (per sensor) at the first stage, followed up with cluster-based nonparametric permutation testing at the second level. This testing was conducted across all participants and the entire frequency range (4–100 Hz) and used an initial cluster threshold of *p* < 0.05 and 5000 permutations. These methods are described in depth in our recent publications [43, 44].

### 2.8. MEG Source Imaging

Time-frequency resolved source images were computed using the dynamic imaging of coherent sources (DICS) beamformer to image oscillatory activity in the time-frequency windows of interest per participant [45–47]. Following convention, we used task and baseline periods of equal duration and bandwidth for each time-frequency cluster identified in the sensor analysis to derive noise-normalized source power per voxel for each participant. The resulting pseudo-*t* maps represent noise-normalized source power differences (i.e., active versus baseline) per participant and voxel (resolution: 4 × 4 × 4 mm). These maps were then transformed into standardized space and spatially resampled by applying the same transform that was applied to the native space structural images per participant.

### 2.9. Whole-Brain Statistics

To probe whole-brain associations between neural oscillatory power serving abstract reasoning and psychosocial distress, we computed voxel-wise correlations between psychosocial distress and spectrally specific maps of neural oscillatory activity [27, 48–50]. Pseudo-*t* values were extracted from the peak voxel of each significant cluster in the resulting maps (i.e., the voxel with the highest statistical value per cluster) in each participant. To account for multiple comparisons, a significance threshold of *p* < 0.005 was used for the identification of significant clusters in all whole-brain statistical maps, accompanied by a cluster (*k*) threshold of at least 25 contiguous voxels (i.e., 1600 mm^3^ of brain tissue) based on the theory of Gaussian random fields [51–53]. All whole-brain statistical analyses were computed using a custom function in MATLAB (MathWorks, Natick, Massachusetts), and other statistical analyses were conducted in IBM SPSS v.25 and Mplus v.8.6. Participant-level oscillatory maps containing significant artifacts were excluded from the correlational analyses.

### 2.10. Data Availability Policy

Requests for data can be fulfilled via the corresponding author. Deidentified data has been made available to the public through the Collaborative Informatics and Neuroimaging Suite (COINS; http://coins.trendscenter.org) database.

## 3. Results

### 3.1. Participant Characteristics and Behavioral Results

Of the 81 participants, 12 were excluded due to poor performance (i.e., accuracy < 60% correct, *n* = 6) on the abstract reasoning task and/or artifactual MEG data (*n* = 5), and one was excluded due to incomplete data on the NIH Toolbox Emotion Battery. Thus, the remaining 69 participants successfully completed the abstract reasoning task and had data on all measures included in the psychosocial distress index. The final sample had a mean age of 44.77 years (SD = 13.66) and a range of 20.22 to 66.89 years. Participants had an average psychosocial distress index of -0.03 (SD = 0.97). Overall, participants performed well on the abstract reasoning task in terms of accuracy (mean = 84.7%, SD = 8.5%) and reaction time (mean = 1991.1 ms, SD = 294.6 ms).

### 3.2. Neural Oscillatory Responses

**Figure 3:**
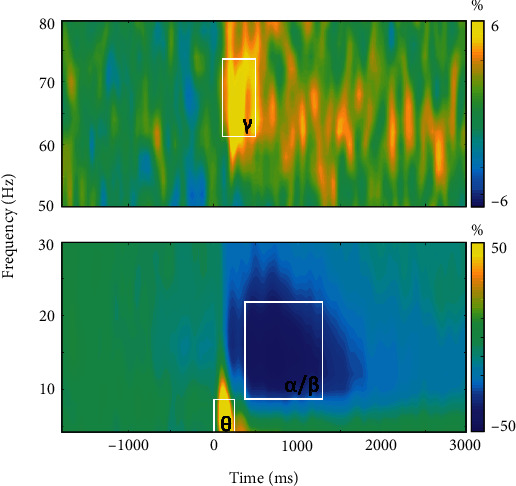
Neural oscillatory responses to the abstract reasoning task. Grand-averaged time-frequency spectrograms of MEG sensors exhibiting one or more significant oscillatory responses. Shown from top to bottom: gamma (62-74 Hz, 175-500 ms), alpha/beta (8-22 Hz, 400-1300 ms), and theta (4-8 Hz, 0-250 ms). Each spectrogram displays frequency (Hz) on the *y*-axis and time (ms) on the *x*-axis. Signal power data are expressed as a percent difference from the baseline period (-1800 to -800 ms) with color legends shown to the right of each spectrogram.

We observed robust neural oscillatory responses in three temporally and spectrally defined windows in response to the abstract reasoning task (Figure 3). These included statistically significant increases in power relative to the baseline period in the theta band (0–250 ms; 4–8 Hz), a decrease in power in the alpha/beta band (400–1300 ms; 8–22 Hz), and an increase in power in the gamma band (175–500 ms; 62–74 Hz). All responses were significant at *p* < 0.005 following multiple comparisons correction using nonparametric permutation testing.

### 3.3. Whole-Brain Correlations with Psychosocial Distress

**Figure 4:**
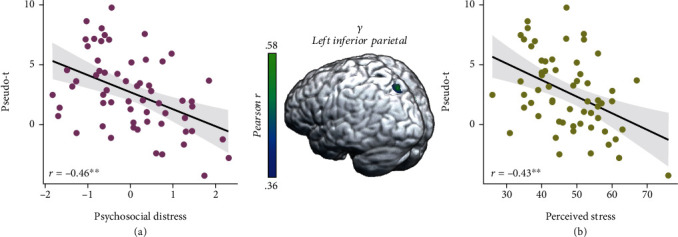
Greater psychosocial distress and perceived stress are associated with blunted gamma activity in the left inferior parietal. (a) Extracted pseudo-*t* values from the peak voxel in the left inferior parietal illustrate the significant relationship between psychosocial distress (*x*-axis) and weaker oscillatory gamma power (pseudo-*t*, *y*-axis) serving abstract reasoning (*r* = −0.46, *p* < 0.005). (b) Perceived stress was also associated with weaker gamma power in the left inferior parietal (*y*-axis; *r* = −0.43, *p* < 0.005). The gray shaded area depicts the standard error of the mean.

**Figure 5:**
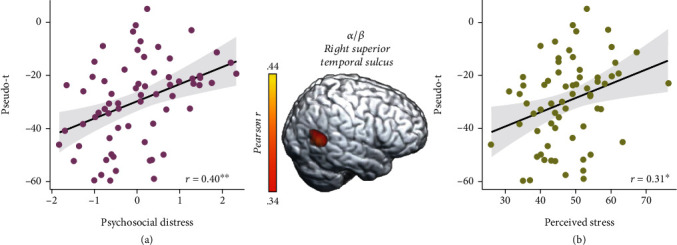
Greater psychosocial distress and perceived stress are inversely correlated with alpha/beta oscillations in the right superior temporal sulcus. (a) Extracted pseudo-*t* values from the peak voxel in the right superior temporal sulcus (image in middle) illustrate the significant relationship between higher psychosocial distress (*x*-axis) and weaker oscillatory alpha/beta responses (pseudo-*t*, *y*-axis) serving abstract reasoning (*r* = 0.40, *p* < 0.005). (b) Greater perceived stress was also associated with weaker alpha/beta oscillations (i.e., less negative) in the right superior temporal sulcus (*y*-axis; *r* = 0.31, *p* = 0.010). The gray shaded area depicts the standard error of the mean.

These three time-frequency windows were imaged for each participant using a beamforming approach. To address our primary hypotheses, these whole-brain, voxel-wise images of theta, alpha/beta, and gamma oscillatory activity were correlated with our psychosocial distress index. These whole-brain correlations revealed that greater psychosocial distress was associated with weaker oscillatory gamma responses during abstract reasoning in the left inferior parietal cortex (*r* = −0.46, *p* < 0.005; Figure 4(a)). Interestingly, gamma oscillatory activity in this inferior parietal region was also associated with higher *T*-scores from the perceived stress scale of the NIH Toolbox Emotion Battery (*r* = −0.43, *p* < 0.005; Figure 4(b)). Additionally, greater psychosocial distress was associated with blunted alpha/beta oscillations in the right superior temporal sulcus (STS, *r* = −0.40, *p* < 0.005; Figure 5(a)), and blunted alpha/beta oscillations in this region were also associated with greater perceived stress (*r* = 0.31, *p* = 0.01; Figure 5(b)). No relationships between theta oscillations and psychosocial distress were detected.

### 3.4. Oscillatory Activity Mediates the Relationship between Perceived Stress and Psychosocial Distress

**Figure 6:**
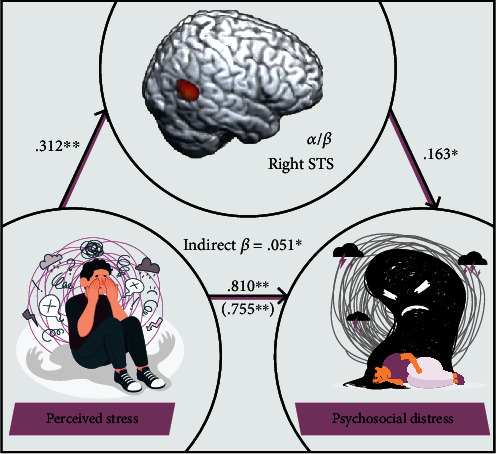
Alpha/beta power in the right superior temporal sulcus partially mediates the relationship between perceived stress and psychosocial distress. Mediation analysis revealed that the relationship between perceived stress and psychosocial distress was partially mediated by alpha/beta power in the right STS. The value in parentheses reflects the total effect of perceived stress on psychosocial distress, which remained statistically significant after adjusting for alpha/beta oscillatory power in the right STS. Significant indirect effects identified by bias-corrected bootstrapped confidence intervals are listed in the center of the model. Standardized regression coefficients are displayed. ⁣^∗^*p* < 0.05; ⁣^∗∗^*p* < 0.01.

Finally, to investigate whether gamma oscillations in the left inferior parietal cortex and alpha/beta oscillations in the right STS separately mediate the relationship between perceived stress and psychosocial distress, we regressed psychosocial distress onto oscillatory gamma power in the left inferior parietal cortex and alpha/beta power in the right STS and found that the strength of gamma oscillations in the left inferior parietal cortex did not significantly mediate the relationship between perceived stress and psychosocial distress. In contrast, we did find that participants with weaker alpha/beta oscillations (i.e., less negative) in the right STS tended to experience greater psychosocial distress (*F* (1, 66) = 11.60, *p* = 0.010). To investigate whether these alpha/beta oscillations mediated the relationship between perceived stress and psychosocial distress, a mediation analysis was conducted [54], with indirect effects estimated using bootstrapping [55]. Our results (Figure 6 and Table 1) indicated a partial mediation of the relationship between perceived stress and psychosocial distress by alpha/beta oscillatory power in the right STS, which survived bootstrapping of 5000 samples (95% CI: 0.001 through 0.013). Importantly, these results suggest that oscillatory alpha/beta responses in the right STS partially drive the effects of perceived stress on psychosocial distress.

## 4. Discussion

In the present study, we investigated the relationship between a transdiagnostic psychosocial distress index and the neural oscillatory dynamics serving abstract reasoning among a sample of healthy adults. Our key findings were that both greater psychosocial distress and higher perceived stress scaled with weaker gamma oscillations in the left inferior parietal cortices and weaker alpha/beta oscillations in the right STS. Further, we found that alpha/beta activity in the right STS mediated the relationship between perceived stress and psychosocial distress. These results corroborate prior work using the stress-diathesis framework, which has linked elevated levels of stress with the presence of greater psychopathological symptomatology [4, 10, 56]. In particular, such work has shown that an individual's environmental context (e.g., stress) is crucial for understanding psychopathological influences across multiple units of analysis, extending from the intricate neural circuitry serving higher-order cognition to self-reported symptomatology and beyond [57, 58]. This environmental context is essential for understanding how one's vulnerabilities or diathesis may remain dormant until activated by some extraneous stressor [58–62].

In terms of task-related neural oscillatory responses, we identified robust oscillatory activity in three distinct frequency bins, including an increase in oscillatory theta (4-8 Hz), a decrease in alpha/beta (8-22 Hz), and an increase in gamma (62-74 Hz) oscillations during the performance of the abstract reasoning task. Source estimation of these oscillatory responses revealed multispectral responses in the frontoparietal and occipital cortices, which is consistent with prior work from our laboratory that used the same task in separate study populations [27, 38]. Further, these findings are in line with the P-FIT model, which posits that the involvement of the frontal and parietal regions are essential for G*f*, with occipital cortices being particularly important for sensory processing [63, 64]. Notably, our results indicate that regionally specific alpha/beta and gamma oscillations serving G*f* are associated with both psychosocial distress and perceived stress. Specifically, those with elevated psychosocial distress and perceived stress had blunted oscillatory alpha/beta and gamma oscillations in association cortices that are crucial for higher-order cognitive function, broadly corroborating the P-FIT [65–67]. In fact, prior studies have suggested that the left inferior parietal cortex is integral to the cognitive mechanisms serving G*f*, which rely on a multiple-demand system including cognitive control and cognitive integration [27, 28, 38, 68, 69]. The inferior parietal cortex participates in a diverse range of cognitive processes, including attention processing and social cognition given its role as a heteromodal association region across a variety of networks involved in multiple cognitive operations [70–72]. Modular segregation and increasing circuit efficiency are central to improved synchronization and information integration in the association cortices, and our results suggest that these intricate networks can be perturbed by perceived stress. Such alterations may reflect the regulatory role that glucocorticoid stress hormones play in synaptic pruning, which can lead to the excessive and irreversible loss of synapses [1, 73–76]. However, further research is needed to support this hypothesis.

Beyond the parietal cortices, we found that greater psychosocial distress and greater perceived stress were associated with attenuated alpha/beta oscillations in the right STS and that these neural responses partially mediated the relationship between perceived stress and psychosocial distress. This is particularly interesting given the diverse role of the STS in higher-order cognition and social behaviors, including complex perceptual, attentional, and linguistic functions [63, 77–80], as well as understanding the actions of others and attributing mental states such as emotions and desires to oneself and to other people [81–85]. Aberrations in both neural activity and cortical structure within the STS have been associated with emotional dysregulation and executive dysfunction across an array of psychiatric disorders [86–88]. Deciphering the potential role that stress may play in altering the neural dynamics in such regions should be a focus of future work, as this could lead to a greater understanding of the interactions between psychosocial distress and psychopathology.

Before closing, it is important to acknowledge some of the limitations of this work. First, we relied on participants to self-report their substance use history and whether they had been diagnosed with a neurological or major psychiatric condition, and thus, we cannot fully rule out whether participants met the diagnostic criteria for these conditions. Further, the cross-sectional design of the study limits some of the conclusions that we can draw from our results, and thus, future studies should consider utilizing longitudinal designs to investigate individualized trajectories of stress-related alterations in cortical oscillatory activity. Such work could strengthen the conclusions identified in the present study and further aid in mapping the stress-induced perturbations to the central nervous system that regulates cognitive and emotional health. Second, we focused solely on an abstract reasoning task, but future work should examine the relationship between stress, psychosocial distress, and tasks that probe other domains of cognition such as working memory and cognitive control. Finally, our study sample was restricted to relatively healthy adults, and thus, these results may not generalize to the overall population. Future studies should identify the relationship between stress and oscillatory activity in clinical samples, such as those with depression and substance- and trauma-related disorders, among others.

While the human body can adapt to moderate stressors by continuously monitoring the environment and readjusting multiple physiological parameters to meet the present demands, such homeostatic balances may be perturbed depending on the frequency, magnitude, and duration of the stressors an individual experiences [89–98]. The National Institute of Mental Health's RDoC framework quantifies a sustained threat construct through various units of analysis that index the specific dimensions of complex behaviors related to chronic stress [56, 91, 99–103], given the established relationship between chronic stress and psychiatric disorders including depression, anxiety, posttraumatic stress disorder (PTSD), and substance use disorders [2, 14, 104–114]. This alone underscores the importance of elucidating the impact of perceived stress on the intricate neural circuitry and dynamics serving higher-order cognition, which we accomplished in the present study. In conclusion, we identified a relationship between attenuated oscillatory activity in the left inferior parietal and right STS and elevated psychosocial distress and perceived stress. Importantly, we also found that alpha/beta activity in the right STS partially mediated the relationship between perceived stress and psychosocial distress. These findings reinforce and expand upon the extant literature suggesting that there is a distributed network serving abstract reasoning capabilities, and more broadly, G*f*. Further, our findings demonstrate that those with elevated psychosocial distress and perceived stress exhibit altered neural oscillatory dynamics in association cortices, which have been frequently linked with executive dysfunction and emotion dysregulation related to preclinical psychopathological symptoms. Dimensional approaches such as those applied in the present study can be harnessed in future work to inform and potentially guide efficacious interventions that may effectively prevent and reduce suffering associated with stress-related psychopathology. Specifically, future studies should investigate the degree to which neuromodulatory techniques such as transcranial alternating current stimulation (tACS) and transcranial magnetic stimulation (TMS) can target stress-related impairments in the alpha/beta and gamma dynamics serving abstract reasoning to mitigate preclinical symptoms of psychosocial distress, and further, prevent the emergence of more severe anxiety and depressive disorders.

## Figures and Tables

**Table 1:** Mediation analysis underlying regressions showing partial mediation of the relationship between perceived stress and psychosocial distress through the right STS.

Model	*b*	SE	*t*	*β*	*F*	*R* ^2^	95% CI
*Simple regression of α/β power in the right STS on perceived stress*							
Intercept	-55.388	9.879	-5.606		7.023	0.098	[-75.118, -35.658]
Perceived stress	0.539	0.203	2.650	0.312			[0.133, 0.946]
*Simple regression of psychosocial distress on perceived stress*							
Intercept	-4.054	0.362	-11.184		128.167	0.652	[-4.778, -3.331]
Perceived stress	0.085	0.008	11.321	0.810			[0.070, 0.100]
*Multiple regression of psychosocial distress on perceived stress and α/β power in the right STS*							
Intercept	-3.524	0.447	-7.875		66.175	0.674	[-4.418, -2.630]
*α*/*β* power in the right STS	0.010	0.005	2.170	0.163			[0.001, 0.019]
Perceived stress	0.080	0.008	10.055	0.755			[0.064, 0.096]

Note. STS: superior temporal sulcus.

## Data Availability

Requests for data can be fulfilled via the corresponding author. Deidentified data have been made available to the public through the Collaborative Informatics and Neuroimaging Suite (COINS; http://coins.trendscenter.org) database.

## References

[1] Lupien S. J., McEwen B. S., Gunnar M. R., Heim C. (2009). Effects of stress throughout the lifespan on the brain, behaviour and cognition. *Nature Reviews Neuroscience*.

[2] Lupien S. J., Sasseville M., François N. (2017). The DSM5/RDoC debate on the future of mental health research: implication for studies on human stress and presentation of the signature bank. *Stress*.

[3] Schantell M., Taylor B. K., Mansouri A. (2024). Theta oscillatory dynamics serving cognitive control index psychosocial distress in youth. *Neurobiology of Stress*.

[4] van Heeringen K., Dwivedi Y. (2012). Stress–diathesis model of suicidal behavior. *The Neurobiological Basis of Suicide. Frontiers in Neuroscience*.

[5] Merrick M. T., Ports K. A., Ford D. C., Afifi T. O., Gershoff E. T., Grogan-Kaylor A. (2017). Unpacking the impact of adverse childhood experiences on adult mental health. *Child Abuse & Neglect*.

[6] Dube S. R., Anda R. F., Felitti V. J., Chapman D. P., Williamson D. F., Giles W. H. (2001). Childhood abuse, household dysfunction, and the risk of attempted suicide throughout the life span: findings from the adverse childhood experiences study. *Journal of the American Medical Association*.

[7] Gloger S., Martínez P., Behn A. (2021). Population-attributable risk of adverse childhood experiences for high suicide risk, psychiatric admissions, and recurrent depression, in depressed outpatients. *European Journal of Psychotraumatology*.

[8] Glenn C. R., Cha C. B., Kleiman E. M., Nock M. K. (2017). Understanding suicide risk within the Research Domain Criteria (RDoC) framework: insights, challenges, and future research considerations. *Clinical Psychological Science: A Journal of the Association for Psychological Science*.

[9] Glenn C. R., Kleiman E. M., Cha C. B., Deming C. A., Franklin J. C., Nock M. K. (2018). Understanding suicide risk within the Research Domain Criteria (RDoC) framework: a meta-analytic review. *Depression and Anxiety*.

[10] van Heeringen K., Mann J. J. (2014). The neurobiology of suicide. *Lancet Psychiatry*.

[11] O'Connor R. C., Nock M. K. (2014). The psychology of suicidal behaviour. *Lancet Psychiatry*.

[12] Ross R. A., Foster S. L., Ionescu D. F. (2017). The role of chronic stress in anxious depression. *Chronic Stress*.

[13] Rottenberg J., Gross J. J., Gotlib I. H. (2005). Emotion context insensitivity in major depressive disorder. *Journal of Abnormal Psychology*.

[14] Morris B. H., Rottenberg J. (2015). Heightened reward learning under stress in generalized anxiety disorder: a predictor of depression resistance?. *Journal of Abnormal Psychology*.

[15] Robinson O. J., Overstreet C., Letkiewicz A., Grillon C. (2012). Depressed mood enhances anxiety to unpredictable threat. *Psychological Medicine*.

[16] Gold P. W. (2015). The organization of the stress system and its dysregulation in depressive illness. *Molecular Psychiatry*.

[17] Juruena M. F., Bocharova M., Agustini B., Young A. H. (2018). Atypical depression and non-atypical depression: is HPA axis function a biomarker? A systematic review. *Journal of Affective Disorders*.

[18] Wohleb E. S., Franklin T., Iwata M., Duman R. S. (2016). Integrating neuroimmune systems in the neurobiology of depression. *Nature Reviews Neuroscience*.

[19] Williams L. M. (2016). Precision psychiatry: a neural circuit taxonomy for depression and anxiety. *Lancet Psychiatry*.

[20] Lener M. S., Iosifescu D. V. (2015). In pursuit of neuroimaging biomarkers to guide treatment selection in major depressive disorder: a review of the literature. *Annals of the New York Academy of Sciences*.

[21] Horn J. L., Cattell R. B. (1966). Refinement and test of the theory of fluid and crystallized general intelligences. *Journal of Educational Psychology*.

[22] McEwen B. S., Morrison J. H. (2013). The brain on stress: vulnerability and plasticity of the prefrontal cortex over the life course. *Neuron*.

[23] Crone E. A., Richard Ridderinkhof K. (2011). The developing brain: from theory to neuroimaging and back. *Developmental Cognitive Neuroscience*.

[24] Duncan J. (2013). The structure of cognition: attentional episodes in mind and brain. *Neuron*.

[25] Pahor A., Jaušovec N. (2014). The effects of theta transcranial alternating current stimulation (tACS) on fluid intelligence. *International Journal of Psychophysiology*.

[26] Neubauer A. C., Wammerl M., Benedek M., Jauk E., Jaušovec N. (2017). The influence of transcranial alternating current stimulation (tACS) on fluid intelligence: an fMRI study. *Personality and Individual Differences*.

[27] Taylor B. K., Embury C. M., Heinrichs-Graham E. (2020). Neural oscillatory dynamics serving abstract reasoning reveal robust sex differences in typically-developing children and adolescents. *Developmental Cognitive Neuroscience*.

[28] Taylor B. K., Heinrichs-Graham E., Eastman J. A. (2022). Longitudinal changes in the neural oscillatory dynamics underlying abstract reasoning in children and adolescents. *NeuroImage*.

[29] Heinrichs-Graham E., Walker E. A., Taylor B. K. (2022). Auditory experience modulates fronto-parietal theta activity serving fluid intelligence. *Brain Communications*.

[30] Dietz S. M., Schantell M., Spooner R. K. (2023). Elevated CRP and TNF-*α* levels are associated with blunted neural oscillations serving fluid intelligence. *Brain, Behavior, and Immunity*.

[31] Wascher E., Rasch B., Sänger J. (2014). Frontal theta activity reflects distinct aspects of mental fatigue. *Biological Psychology*.

[32] Cavanagh J. F., Frank M. J. (2014). Frontal theta as a mechanism for cognitive control. *Trends in Cognitive Sciences*.

[33] Başar E., Güntekin B. (2013). Review of delta, theta, alpha, beta, and gamma response oscillations in neuropsychiatric disorders. *Supplements to Clinical Neurophysiology*.

[34] Colgin L. L. (2013). Mechanisms and functions of theta rhythms. *Annual Review of Neuroscience*.

[35] DiStefano C., Zhu M., Mîndrilã D. (2009). Understanding and using factor scores: considerations for the applied researcher. *Practical Assessment, Research, and Evaluation*.

[36] John R. J., McCallum R. S. (2003). Raven progressive matrices. *Handbook of Nonverbal Assessment*.

[37] Penhale S. H., Arif Y., Schantell M. (2024). Healthy aging alters the oscillatory dynamics and fronto-parietal connectivity serving fluid intelligence. *Human Brain Mapping*.

[38] Arif Y., Spooner R. K., Heinrichs-Graham E., Wilson T. W. (2021). High-definition transcranial direct current stimulation modulates performance and alpha/beta parieto-frontal connectivity serving fluid intelligence. *The Journal of Physiology*.

[39] Taulu S., Simola J. (2006). Spatiotemporal signal space separation method for rejecting nearby interference in MEG measurements. *Physics in Medicine and Biology*.

[40] Uusitalo M. A., Ilmoniemi R. J. (1997). Signal-space projection method for separating MEG or EEG into components. *Medical & Biological Engineering & Computing*.

[41] Papp N., Ktonas P. (1977). Critical evaluation of complex demodulation techniques for the quantification of bioelectrical activity. *Biomedical Sciences Instrumentation*.

[42] Kovach C. K., Gander P. E. (2016). The demodulated band transform. *Journal of Neuroscience Methods*.

[43] Wiesman A. I., Christopher-Hayes N. J., Eastman J. A., Heinrichs-Graham E., Wilson T. W. (2021). Response certainty during bimanual movements reduces gamma oscillations in primary motor cortex. *NeuroImage*.

[44] Wiesman A. I., Wilson T. W. (2020). Attention modulates the gating of primary somatosensory oscillations. *NeuroImage*.

[45] Van Veen B. D., van Drongelen W., Yuchtman M., Suzuki A. (1997). Localization of brain electrical activity via linearly constrained minimum variance spatial filtering. *IEEE Transactions on Biomedical Engineering*.

[46] Gross J., Kujala J., Hämäläinen M., Timmermann L., Schnitzler A., Salmelin R. (2001). Dynamic imaging of coherent sources: studying neural interactions in the human brain. *Proceedings of the National Academy of Sciences of the United States of America*.

[47] Dalal S. S., Sekihara K., Nagarajan S. S. (2006). Modified beamformers for coherent source region suppression. *IEEE Transactions on Biomedical Engineering*.

[48] Embury C. M., Wiesman A. I., Proskovec A. L. (2019). Neural dynamics of verbal working memory processing in children and adolescents. *NeuroImage*.

[49] Groff B. R., Wiesman A. I., Rezich M. T. (2020). Age-related visual dynamics in HIV-infected adults with cognitive impairment. *Neurology Neuroimmunology & Neuroinflammation*.

[50] Schantell M., Taylor B. K., Spooner R. K. (2022). Epigenetic aging is associated with aberrant neural oscillatory dynamics serving visuospatial processing in people with HIV. *Aging*.

[51] Poline J. B., Worsley K. J., Holmes A. P., Frackowiak R. S. J., Friston K. J. (1995). Estimating smoothness in statistical parametric maps: variability of p values. *Journal of Computer Assisted Tomography*.

[52] Worsley K. J., Marrett S., Neelin P., Vandal A. C., Friston K. J., Evans A. C. (1996). A unified statistical approach for determining significant signals in images of cerebral activation. *Human Brain Mapping*.

[53] Worsley K. J., Andermann M., Koulis T., MacDonald D., Evans A. C. (1999). Detecting changes in nonisotropic images. *Human Brain Mapping*.

[54] Baron R. M., Kenny D. A. (1986). The moderator–mediator variable distinction in social psychological research: conceptual, strategic, and statistical considerations. *Journal of Personality and Social Psychology*.

[55] Preacher K. J., Hayes A. F. (2004). SPSS and SAS procedures for estimating indirect effects in simple mediation models. *Behavior Research Methods, Instruments & Computers*.

[56] Stewart J. G., Polanco-Roman L., Duarte C. S., Auerbach R. P. (2019). Neurocognitive processes implicated in adolescent suicidal thoughts and behaviors: applying an RDoC framework for conceptualizing risk. *Current Behavioral Neuroscience Reports*.

[57] Steidtmann D., Ingram R. E., Siegle G. J. (2010). Pupil response to negative emotional information in individuals at risk for depression. *Cognition and Emotion*.

[58] Woody M. L., Gibb B. E. (2015). Integrating NIMH Research Domain Criteria (RDoC) into depression research. *Current Opinion in Psychology*.

[59] Gee D. G., Humphreys K. L., Flannery J. (2013). A developmental shift from positive to negative connectivity in human amygdala-prefrontal circuitry. *The Journal of Neuroscience*.

[60] Cole D. A., Ciesla J. A., Dallaire D. H. (2008). Emergence of attributional style and its relation to depressive symptoms. *Journal of Abnormal Psychology*.

[61] Lakdawalla Z., Hankin B. L., Mermelstein R. (2007). Cognitive theories of depression in children and adolescents: a conceptual and quantitative review. *Clinical Child and Family Psychology Review*.

[62] Kellough J. L., Beevers C. G., Ellis A. J., Wells T. T. (2008). Time course of selective attention in clinically depressed young adults: an eye tracking study. *Behaviour Research and Therapy*.

[63] Jung R. E., Haier R. J. (2007). The Parieto-frontal integration theory (P-FIT) of intelligence: converging neuroimaging evidence. *The Behavioral and Brain Sciences*.

[64] Basten U., Hilger K., Fiebach C. J. (2015). Where smart brains are different: a quantitative meta-analysis of functional and structural brain imaging studies on intelligence. *Intelligence*.

[65] Christoff K., Prabhakaran V., Dorfman J. (2001). Rostrolateral prefrontal cortex involvement in relational integration during reasoning. *NeuroImage*.

[66] Duncan J. (2005). Frontal lobe function and general intelligence: why it matters. *Cortex*.

[67] Kroger J. K., Sabb F. W., Fales C. L., Bookheimer S. Y., Cohen M. S., Holyoak K. J. (2002). Recruitment of anterior dorsolateral prefrontal cortex in human reasoning: a parametric study of relational complexity. *Cerebral Cortex*.

[68] Proskovec A. L., Wiesman A. I., Wilson T. W. (2019). The strength of alpha and gamma oscillations predicts behavioral switch costs. *Neuroimage*.

[69] Duncan J., Assem M., Shashidhara S. (2020). Integrated intelligence from distributed brain activity. *Trends in Cognitive Sciences*.

[70] Numssen O., Bzdok D., Hartwigsen G. (2021). Functional specialization within the inferior parietal lobes across cognitive domains. *eLife*.

[71] Bzdok D., Hartwigsen G., Reid A., Laird A. R., Fox P. T., Eickhoff S. B. (2016). Left inferior parietal lobe engagement in social cognition and language. *Neuroscience and Biobehavioral Reviews*.

[72] Bzdok D., Schilbach L., Vogeley K. (2012). Parsing the neural correlates of moral cognition: ALE meta-analysis on morality, theory of mind, and empathy. *Brain Structure & Function*.

[73] Gabard-Durnam L., McLaughlin K. A. (2020). Sensitive periods in human development: charting a course for the future. *Current Opinion in Behavioral Sciences*.

[74] Sydnor V. J., Larsen B., Bassett D. S. (2021). Neurodevelopment of the association cortices: patterns, mechanisms, and implications for psychopathology. *Neuron*.

[75] Liston C., Gan W. B. (2011). Glucocorticoids are critical regulators of dendritic spine development and plasticity in vivo. *National Academy of Sciences of the United States of America*.

[76] Liston C., Cichon J. M., Jeanneteau F., Jia Z., Chao M. V., Gan W. B. (2013). Circadian glucocorticoid oscillations promote learning-dependent synapse formation and maintenance. *Nature Neuroscience*.

[77] Fedorenko E., Nieto-Castañon A., Kanwisher N. (2012). Lexical and syntactic representations in the brain: an fMRI investigation with multi-voxel pattern analyses. *Neuropsychologia*.

[78] Jung J., Cloutman L. L., Binney R. J., Lambon Ralph M. A. (2017). The structural connectivity of higher order association cortices reflects human functional brain networks. *Cortex*.

[79] Karim A. K. M. R., Proulx M. J., de Sousa A. A., Likova L. T. (2021). Neuroplasticity and crossmodal connectivity in the normal, healthy brain. *Psychology & Neuroscience*.

[80] Barraclough N. E., Xiao D., Baker C. I., Oram M. W., Perrett D. I. (2005). Integration of visual and auditory information by superior temporal sulcus neurons responsive to the sight of actions. *Journal of Cognitive Neuroscience*.

[81] Paulus F. M., Müller-Pinzler L., Jansen A., Gazzola V., Krach S. (2015). Mentalizing and the role of the posterior superior temporal sulcus in sharing others’ embarrassment. *Cerebral Cortex*.

[82] Jankowski K. F., Takahashi H. (2014). Cognitive neuroscience of social emotions and implications for psychopathology: examining embarrassment, guilt, envy, and schadenfreude. *Psychiatry and Clinical Neurosciences*.

[83] Takahashi H., Matsuura M., Koeda M. (2008). Brain activations during judgments of positive self-conscious emotion and positive basic emotion: pride and joy. *Cerebral Cortex*.

[84] Redcay E., Schilbach L. (2019). Using second-person neuroscience to elucidate the mechanisms of social interaction. *Nature Reviews Neuroscience*.

[85] Redcay E., Velnoskey K. R., Rowe M. L. (2016). Perceived communicative intent in gesture and language modulates the superior temporal sulcus. *Human Brain Mapping*.

[86] Lee J., Lee D., Namkoong K., Jung Y. C. (2020). Aberrant posterior superior temporal sulcus functional connectivity and executive dysfunction in adolescents with internet gaming disorder. *Journal of Behavioral Addictions*.

[87] Wang Y., Jiang P., Tang S. (2021). Left superior temporal sulcus morphometry mediates the impact of anxiety and depressive symptoms on sleep quality in healthy adults. *Social Cognitive and Affective Neuroscience*.

[88] Li N., Jin D., Wei J., Huang Y., Xu J. (2022). Functional brain abnormalities in major depressive disorder using a multiscale community detection approach. *Neuroscience*.

[89] Malter Cohen M., Tottenham N., Casey B. J. (2013). Translational developmental studies of stress on brain and behavior: implications for adolescent mental health and illness?. *Neuroscience*.

[90] Shonkoff J. P., Boyce W. T., McEwen B. S. (2009). Neuroscience, molecular biology, and the childhood roots of health disparities: building a new framework for health promotion and disease prevention. *Journal of the American Medical Association*.

[91] Sheth C., McGlade E., Yurgelun-Todd D. (2017). Chronic stress in adolescents and its neurobiological and psychopathological consequences: an RDoC perspective. *Chronic Stress*.

[92] McEwen B. S. (2006). Protective and damaging effects of stress mediators: central role of the brain. *Dialogues in Clinical Neuroscience*.

[93] McEwen B. S. (1998). Stress, adaptation, and disease: allostasis and allostatic load. *Annals of the New York Academy of Sciences*.

[94] Seeman T. E., Singer B. H., Rowe J. W., Horwitz R. I., McEwen B. S. (1997). Price of adaptation--allostatic load and its health consequences. MacArthur studies of successful aging. *Archives of Internal Medicine*.

[95] Koob G. F., Le Moal M. (2001). Drug addiction, dysregulation of reward, and allostasis. *Neuropsychopharmacology*.

[96] Karlamangla A. S., Singer B. H., McEwen B. S., Rowe J. W., Seeman T. E. (2002). Allostatic load as a predictor of functional decline. MacArthur studies of successful aging. *Journal of Clinical Epidemiology*.

[97] Geronimus A. T., Hicken M., Keene D., Bound J. (2006). “Weathering” and age patterns of allostatic load scores among blacks and whites in the United States. *American Journal of Public Health*.

[98] Juster R. P., McEwen B. S., Lupien S. J. (2010). Allostatic load biomarkers of chronic stress and impact on health and cognition. *Neuroscience & Biobehavioral Reviews*.

[99] Insel T., Cuthbert B., Garvey M. (2010). Research Domain Criteria (RDoC): toward a new classification framework for research on mental disorders. *American Journal of Psychiatry*.

[100] Cuthbert B. N., Insel T. R. (2013). Toward the future of psychiatric diagnosis: the seven pillars of RDoC. *BMC Medicine*.

[101] Cuthbert B. N. (2014). The RDoC framework: facilitating transition from ICD/DSM to dimensional approaches that integrate neuroscience and psychopathology. *World Psychiatry*.

[102] Pacheco J., Garvey M. A., Sarampote C. S., Cohen E. D., Murphy E. R., Friedman-Hill S. R. (2022). Annual research review: the contributions of the RDoC research framework on understanding the neurodevelopmental origins, progression and treatment of mental illnesses. *Journal of Child Psychology and Psychiatry*.

[103] Clark L. A., Cuthbert B., Lewis-Fernández R., Narrow W. E., Reed G. M. (2017). Three approaches to understanding and classifying mental disorder: ICD-11, DSM-5, and the National Institute of Mental Health’s Research Domain Criteria (RDoC). *Psychological Science in the Public Interest*.

[104] Dvir Y., Ford J. D., Hill M., Frazier J. A. (2014). Childhood maltreatment, emotional dysregulation, and psychiatric comorbidities. *Harvard Review of Psychiatry*.

[105] Kim E. Y., Miklowitz D. J., Biuckians A., Mullen K. (2007). Life stress and the course of early-onset bipolar disorder. *Journal of Affective Disorders*.

[106] Brady K. T., Sinha R. (2005). Co-occurring mental and substance use disorders: the neurobiological effects of chronic stress. *The American Journal of Psychiatry*.

[107] Farb N. A. S., Irving J. A., Anderson A. K., Segal Z. V. (2015). A two-factor model of relapse/recurrence vulnerability in unipolar depression. *Journal of Abnormal Psychology*.

[108] Muhtadie L., Johnson S. L. (2015). Threat sensitivity in bipolar disorder. *Journal of Abnormal Psychology*.

[109] Weiss R. B., Stange J. P., Boland E. M. (2015). Kindling of life stress in bipolar disorder: comparison of sensitization and autonomy models. *Journal of Abnormal Psychology*.

[110] Farmer A. S., Kashdan T. B. (2015). Stress sensitivity and stress generation in social anxiety disorder: a temporal process approach. *Journal of Abnormal Psychology*.

[111] McLaughlin K. A., Rosen M. L., Kasparek S. W., Rodman A. M. (2022). Stress-related psychopathology during the COVID-19 pandemic. *Behaviour Research and Therapy*.

[112] McLaughlin K. A., Sheridan M. A. (2016). Beyond cumulative risk: a dimensional approach to childhood adversity. *Current Directions in Psychological Science*.

[113] McLaughlin K. A., Koenen K. C., Bromet E. J. (2017). Childhood adversities and post-traumatic stress disorder: evidence for stress sensitisation in the World Mental Health Surveys. *British Journal of Psychiatry*.

[114] McLaughlin K. A., Greif Green J., Gruber M. J., Sampson N. A., Zaslavsky A. M., Kessler R. C. (2012). Childhood adversities and first onset of psychiatric disorders in a national sample of US adolescents. *Archives of General Psychiatry*.

